# The Impact of the Coronavirus Disease (COVID-19) Pandemic on Nurses’ Turnover Intention: An Integrative Review

**DOI:** 10.3390/nursrep11040075

**Published:** 2021-10-19

**Authors:** Rawaih Falatah

**Affiliations:** College of Nursing, King Saud University, Riyadh 11362, Saudi Arabia; rfalatah@ksu.edu.sa; Tel.: +966-11-8051864

**Keywords:** COVID-19, nurses’ turnover, integrative review

## Abstract

The COVID-19 pandemic has increased the demand and workload on nurses. In addition, the number of critical cases, the uncertainty about the disease, and the incidence rate of death from the disease impose a psychological stress on nurses. Considering the alarming issues of stress, burnout, and turnover among nurses even before the pandemic, the pandemic might have amplified such issues. Thus, the impact of the COVID-19 pandemic on nurses’ turnover and turnover intention warrants investigation. The aim of this review is to appraise and integrate the current pre- and post-coronavirus disease (COVID-19) literature on nurse turnover, published between 2016 and 2021. Forty-three studies on nurses’ turnover intention were appraised and synthesized. The reviewed literature suggested that nurses’ turnover intention increased significantly after the COVID-19 pandemic. Post-COVID-19-pandemic studies focused more on predicting nurses’ turnover intention through the pandemic’s negative impact on the nurses’ psychological wellbeing. The findings of this review should be considered by nurse managers and leaders in the development of policies and programs to reduce the negative impact of COVID-19 on nurse retention.

## 1. Introduction

Nurse turnover has been defined as a global healthcare-system issue [1]. It has been defended as voluntary and early termination of nurses’ employment [2]. Nurse turnover could be either organizational or professional, with the latter being the most consequential because of its contribution to the preexisting nurse shortage [3]. In the literature, studies that measured the actual organizational and professional nurse turnover are limited; however, many studies have evaluated nurses’ turnover intention. In addition, nurses’ turnover intention has been defined in the literature as the most accurate predictor of actual nurse turnover [4].

Many terms have been used in the nursing literature that are synonymous with nurses’ turnover intention, such as intention to leave, intention to quit, intention to stay, risk of quitting, and job retention intention [5,6,7]. Although increasing attention has been paid to the difference between organizational and professional turnover intentions, the lack of consistency of the definition and measurement of organizational turnover, professional turnover, organizational turnover intention, and professional turnover intention is still evident in the nursing literature [8]. Nonetheless, nurses’ turnover intention has been widely defined as “an individual’s perceived probability of permanently leaving the employing organization in the near future” [1].

Turnover intention has been linked to several adverse outcomes such as medication error, falls, and pressure injuries [9]. Moreover, it has been linked to increased healthcare system costs due to its impact on both financial and time resources [10]. Thus, numerous studies have been conducted to identify nurses’ turnover intention predictors and provide useful information for the development of remedial programs to lower nurses’ turnover intention rates. Among the identified predictors are job satisfaction, job commitment, stress, anxiety, and burnout [11]. Historically, the prementioned predictors were found to be impacted by major crises such as pandemics [12]. Hence, the coronavirus disease (COVID-19) pandemic can impact these predictors and therefore impact nurses’ turnover and turnover intention.

The first case of COVID-19 was reported in Wuhan City, Hubei Province of China, and the World Health Organization (WHO) office was informed about the novel disease on 31 December 2019 [13]. Currently, the number of reported cases is 186 million, and the number of deaths exceeds 4 million [13]. The pandemic caused vast closures and travel bans in the global attempt to control it, which has had economic and psychological impacts on the public. In addition, it imposed an unprecedented demand on healthcare systems and workers worldwide [14]. Studies have reported that COVID-19 has impacted healthcare workers physically and psychologically, many of whom reported fears of contracting the disease or infecting a loved one [15].

Nurses are the largest group in the healthcare team and have the longest contact time with patients [9]. Thus, the pandemic increased the demand and workload on nurses in an extreme work environment. The number of critical cases, the uncertainty about the disease, and the incidence of death from the disease impose a psychological stress on nurses [16]. Considering the alarming issues of stress, burnout, and turnover among nurses even before the pandemic [10], the pandemic might have amplified the issue. Thus, the impact of the COVID-19 pandemic on the rates and predictors of nurses’ turnover and turnover intention must be investigated by appraising and integrating the current literature on nurse turnover before and after the pandemic.

### 1.1. Aim

The aim of this review was to appraise and integrate the current turnover literature, published between 2016 and 2021, by using Whittemore and Knafl’s integrative review method [17]. The review was aimed at answering the following questions: How is nurse turnover defined and measured? What are the differences in turnover and turnover intention rates before and after the COVID-19 pandemic? What are the predictors of nurse turnover before and after the COVID-19 pandemic?

### 1.2. Methods

The integrative review method was described by Whittemore and Knalfl as the most comprehensive and inclusive review method because it enables reviewers to include varied articles, including empirical qualitative and quantitative studies and theoretical reports. Thus, this method was utilized to minimize the review bias and increase the rigor of the study. Whittemore and Knalfl identified six phases of their review method, namely problem identification, literature search, data evaluation, data analysis, and presentation of conclusions. Since the study is a review of the literature, it did not require IRB approval.

### 1.3. Problem Identification

The problem identification stage is the first phase in the review technique and makes the following phases easier to perform. The goal of this phase is to set the focus and bounds to the otherwise vast, complex, and ambiguous review. This goal is achieved by identifying the target concepts, population, healthcare problem, and sample frame [17]. Indeed, nurse turnover is a problem that has impacted healthcare systems and outcomes worldwide. The impacts of COVID-19 on nurses’ physical and psychological wellbeing are evident in the literature [18]. Thus, we hypothesized that COVID-19 will amplify the problem of nurse turnover. The target concepts for this review are nurses’ turnover and turnover intention, and the COVID-19 pandemic.

### 1.4. Literature Search

In accordance with Whittemore and Knafl’s method, the objective of the literature search phase is to incorporate as many suitable primary sources as possible within a predefined frame [17]. The use of various search techniques is recommended, and purposive sampling may be used if necessary. Transparency and explanation of each sample decision are critical components of this phase [17].

**Figure 1:**
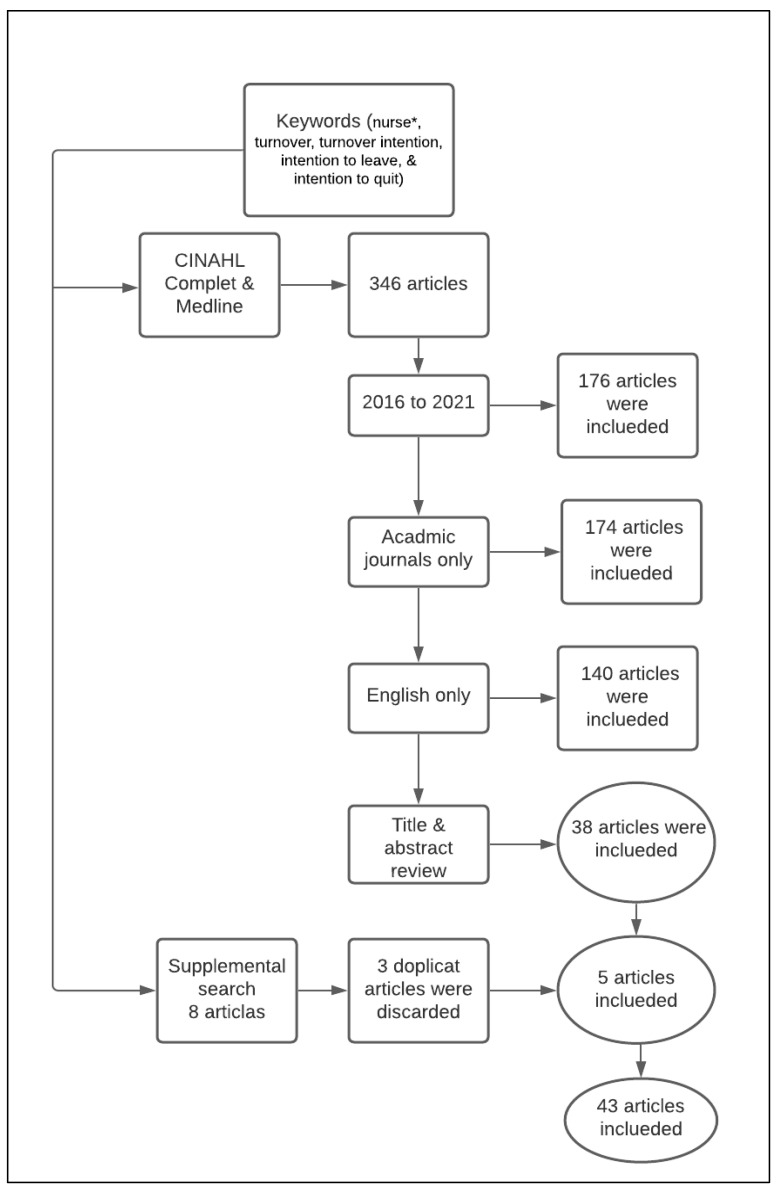
Flow chart of literature search. * To include (nurse, nurses, & nursing). CINAHL: Cumulative Index of Nursing and Allied Health Literature.

For this review, the literature search was conducted using a preformulated search strategy to identify current articles on pre- and post-COVID-19 pandemic nurses’ turnover. Initially, the Cumulative Index of Nursing and Allied Health Literature (CINAHL) and Medline databases were searched, followed by a supplemental search on Google Scholar. The databases were accessed using the Saudi Digital Library. The keywords used included “nurse*,” “turnover,” “turnover intention,” “intention to leave,” and “intention to quit.” The steps of the literature search are presented in Figure 1.

The initial database search yielded 346 articles. Limiting the search to the years between 2016 and 2021 to focus the review on the most recent literature pre-and post-COVID-19 resulted in 176 articles. When the search was additionally limited to academic journals only, the number of articles became 174. Finally, 140 articles remained when the search was limited to English-only articles. The author reviewed the titles of the 140 articles and identified 55 suitable articles that met the inclusion criteria (Table 1). After reviewing the abstract of these articles based on the inclusion criteria, 38 were selected. After obtaining the sample, an independent expert was consulted about the inclusion criteria, the search strategy, and the included articles. She suggested a supplemental Google Scholar search and five more articles were included in the review after the supplemental search. A total of 43 articles were included in this review. Only primary peer reviewed articles were included in this review and all the grey literature items were excluded. Articles that were discussing turnover in other professions and not including nursing were excluded. For example, articles that were examining turnover among physicians were excluded, however articles that were examining turnover among physicians and nurses were included.

### 1.5. Data Evaluation

Owing to the different research methods of the primary sources, evaluating the quality of the primary sources for an integrated review may be difficult. As a result, the data evaluation step in an integrative review is determined by the sample frame used [17]. For example, if the reviewed article includes qualitative and quantitative studies, two different appraisal tools must be used to evaluate the studies. All the studies included in this review used a quantitative research method, and most were cross-sectional studies. Thus, a modified Johns Hopkins Nursing Evidence-Based Practice Research Evidence Appraisal Tool was used to evaluate the reviewed studies. As all the studies did not include a control group, the items used to evaluate the use of a control group were eliminated, and 12 questions were used to appraise the studies. A one-point score was assigned to each of the 12 appraisal items, and the quality score ranged from 12 to 0. The score was divided into three levels as follows: from 12 to 9, high quality; from 8 to 5, good quality; and from 4 to 0, low quality. The quality assessment is summarized in Table 2.

Most of the studies rated as high quality in the quality assessment obtained 11 points (*n* = 16), followed by those who obtained 12 points (*n* = 10), 10 points (*n* = 8); and 9 points (*n* = 7). Only two articles obtained 8 points and were rated as good quality. More than half of the reviewed articles (*n* = 24) scored 0 in the item related to the use of current literature, as more than half of the used sources in these articles were published >5 years from the year of publication. Instrument validity was not discussed on 18 of the reviewed articles. Moreover, turnover instrument reliability was not reported in seven of the reviewed articles. Similarly, the survey response rate was not reported in seven of the reviewed articles.

## 2. Results

### 2.1. Data Analysis

The goal of the data analysis phase is to provide an answer to the preidentified problem while facilitating a thorough understanding of the review topic. Data analysis involves organizing, classifying, categorizing, and summarizing information from primary sources to arrive at a methodical and creative conclusion. The steps of the data analysis in the integrative review technique include data reduction, data display, data comparison, conclusion drafting, and validation [17].

### 2.2. Data Reduction and Display

In this phase, the articles are divided into subgroups according to, for example, demographics or countries, or historical events. The data are then taken from primary sources and organized into a comprehensible structure such as a matrix or spreadsheet. The data are then compiled into a presentation based on certain factors or categories [17]. This review aimed to evaluate the impact of the COVID-19 pandemic on nurse turnover. Thus, the reviewed studies were divided into two subgroups, pre- and post-COVID-19 studies. Data, including author and year, purpose, time (pre- or post-COVID-19), method, variable, turnover prevalence, turnover predictors, and turnover outcomes, were extracted using a spreadsheet (Table 3). The outcome column was eliminated from the displayed table because no data were found in any of the articles related to the outcomes of nurse turnover.

### 2.3. Data Comparison

Data comparison is an interactive technique that involves reviewing the data presentation to discover similar themes, patterns, and/or correlations among the primary resources [17]. While the design of most studies was identified as cross-sectional, two of the studies had a longitudinal design [38,42]. No explicit definition of turnover intention was provided in 28 of the reviewed studies. While two of the reviewed studies did not explicitly discuss how turnover intention was measured [6,35], eight studies used a single item (Table 3).

Ten of the 43 reviewed studies were conducted after the COVID-19 pandemic [5,7,12,15,16,18,19,20,21,22]. The aims of 7 of the 10 studies included investigation of the impact of psychological factors such as anxiety, stress, and fear of COVID-19 on nurses’ turnover and turnover intention [5,7,12,15,16,18,21,22]. The other three studies aimed to examine the impact of social support on nurses’ retention intention [7], determine the association between workplace bullying and intention to leave [19], and compare nurse turnover before and after the COVID-19 pandemic [20].

The purposes of the 32 pre-COVID-19 articles can be viewed in Table 3. The purposes included assessing nurses’ turnover intention rates [24,29,38], identifying nurses’ turnover intention factors [10,14,44], and examining the association of turnover intention with variables such as job satisfaction, leadership style, burnout, and work climate [11,23,39,40]. In summary, we can conclude that most post-COVID-19 studies aimed to examine the psychological impacts of the pandemic on nurses’ turnover and turnover intention. By contrast, the pre-COVID-19 studies aimed to examine the association between nurse turnover and varied staff-, profession-, patient-, and organization-related factors.

Two of the 10 post-COVID-19 studies assessed nurses’ intention to stay and retention intention [5,7], which were 2.00 and 3.91, respectively. The rest of the post-COVID-19 studies measured nurses’ turnover intention, and the mean ranged from 2.23 to 3.42 [15,18]. Two of the post-COVID-19 studies measured professional turnover intention and obtained mean values of 1.86 and 2.87 [15,29]. Nashwan and others found a significant increase in turnover intention from a mean of 13.24 to 15.54 [20]. Similarly, a comparative study conducted in Egypt by Said & El-Shafei on 210 nurses from the Zagazig Fever Hospital (ZFH), a COVID-19 triage hospital and 210 nurses from the Zagazig General Hospital (ZGH), which is neither a triage nor an isolation hospital, found significant differences in intention to leave current position (ZFH = 40.0%; ZGH = 30.5%), intention to leave current organization (ZFH = 45.2%; ZGH = 34.3%), and intention to leave the field of nursing (ZFH = 24.8%; ZGH = 10.0%) [12].

Among the 32 pre-COVID-19 studies, 3 measured nurses’ professional turnover, and the rates ranged from 11.2% [38] to 42.2% [14]. On the other hand, nurses’ organizational turnover intention was measured in 29 pre-COVID-19 studies, and the rates ranged from 94% [29] to 14.06% [43]. The mean nurses’ turnover intention pre-COVID-19 ranged from 1.88 [40] to 3.70 [45].

The predictor of job turnover intention post-COVID-19 was dependent on the nurses’ age and work experience. In terms of the factors related to COVID-19, the group with experience in nursing care for patients with COVID-19 infection and those working in COVID-19 divisions had high rates of job turnover intention. Lastly, job engagement and turnover intention appeared to differ depending on the category and type of social support available for the nurses [7]. The identified predictors of turnover intention after the COVID-19 pandemic were; frequency of providing care to patients in the workplace, having taken a course on providing care related to pandemic prevention after starting work, willingness to provide services, and clinical stress [5]. In addition, nurses’ fear of contracting COVID-19 was significantly associated with increased turnover intention [15,18,21]. The factors perceived to increase nurses fear of COVID-19 include lack of experience in nursing care for patients with COVID-19 infection and working in COVID-19 divisions (Kim et al., 2020), deployment in COVID-19 departments [12,20], and age, wherein younger nurses had higher turnover intention [22]. Liaqat and others conducted a study from September 2019 to April 2020 to examine the association between workplace bullying and nurses’ intention to leave the job [19]. They found that most study participants affirmed that work-related bullying was the reason behind their intention to quit their job (59.5%). Many of the study participants affirmed that person-related bullying was present in their workplace and was the reason behind their intention to quit their job (39.7%). Physically intimidating bullying was affirmed by 40.9% of the nurses and was the reason behind their intention to quit their job.

The predictors of turnover intention in the pre-COVID-19 studies included sociodemographic characteristics such as age [35,38], marital status [23,39], nationality [23], and sex [26]. Nurses’ physical health [23,30] and psychological states [23,36] were significant predictors of turnover intention. Moreover, while nurse burnout was significantly positively associated with turnover intention [11,25,32,42], nurses’ job satisfaction was significantly negatively associated with turnover intention [1,6,27,28,37]. Similarly, higher levels of organizational and professional commitments were associated with lower nurses’ turnover intention [4,29,31].

In the reviewed articles, job-related characteristics such as clinical assignment, job demand, passiveness, and workload significantly predicted nurses’ turnover intention [4,11,30]. In addition, while recognition was associated with lower nurses’ turnover intention [3,24,33], workplace bullying was associated with higher nurses’ turnover intention [7,28]. Vévoda, Vévodová, Bubeníková, Kisvetrová, and Ivanová; and Wubetie et al. found that salary is associated with nurses’ turnover intention [2,44]. Furthermore, leadership and supervision support were significantly associated with turnover intention [34,40,47]. All the predictors of nurses’ turnover intention post-COVID-19 are presented in Table 3.

## 3. Discussion

This is the first integrative review study that aimed to examine the prevalence and predictors of nurses’ turnover and turnover intention before and after the COVID-19 pandemic. As in previous studies and reviews, no consensus was reached regarding the definition and measurement of nurses’ turnover and turnover intention [27]. This lack of congruency might be the cause of the observed variation in nurses’ turnover and turnover intention rates. Nonetheless, nurses’ turnover intention is considered high compared with those of other professions [10], and COVID-19 appeared to have increased the mean nurses’ turnover intention rate. This result has been forecasted by many studies and is reported to be due to the social and psychological impacts of COVID-19 [7,18].

The findings of this review have confirmed that post-COVID-19, the most reported nurses’ turnover intentions predictors included fear of the disease, stress, and anxiety. This predictor differs from the pre-COVID-19 turnover intention predictors, which included satisfaction, commitment, and leadership style. This expectation was confirmed in a few studies that supported the evidence of the significant impacts of the COVID-19 pandemic on nurses’ turnover and turnover intention. Hence, more studies are needed to enhance the understanding of the impacts of COVID-19 on nurses’ outcomes, including turnover intention.

## 4. Implication

As per the findings of this review, future research in nursing turnover should be based on clear and explicit theoretical and operational definition of nurses’ turnover and turnover intention. This will improve the consistency across the nurses’ turnover-related literature and provide a more accurate estimation of the proviolence of the problem. Additionally, future research on nurses’ turnover should utilize experimental design to examine the impact of interventions based on the uncovered significant predictors.

In management, nurse leaders and policy makers should build polices and regulation based on the evidence found in the literature to decrease nurses’ turnover. These include reconsidering aspects such as nurses’ workload, pay and benefits, and educational reimbursement. At the front-line management level, nursing mangers should strive to improve staff nurses’ satisfaction through their inclusion in decision making and give them autonomy of their clinical practice. Additionally, during pandemics and crises, nursing mangers should ensure that their staff have adequate social and psychological support systems. Lastly, providing nurses with appreciation and acknowledgment might play an important role in decreasing nurses’ turnover and turnover intention during unpresented crises.

## 5. Limitation

Although this study utilized an integrative review method that enabled the inclusion of studies with varied designs, the lack of a statistical method, which is utilized in meta-analyses and would confirm the statistical significance of the pre- and post-COVID-19 differences in nurses’ turnover, could be considered a limitation. Thus, future reviews in this area should implement meta-analysis review methods that enable the use of statistical methods. Nonetheless, the finding of this review provides a wide appraisal and integration of the current literature on pre- and post-COVID-19 nurses’ turnover intentions.

Another limitation is that this review was conducted by single reviewer, which might impact the rigor of the review. To mitigate this limitation, the author consulted a nursing-management research expert several times during the review.

## 6. Conclusions

The unpresented COVID-19 pandemic has impacted many aspects of people’s lives, of which healthcare services are one of the most impacted. Nurses, being the largest group of healthcare providers who spend the longest time with patients, are among the healthcare groups that are highly impacted by the pandemic. Before the pandemic, nursing was one of the professions with the highest turnover intention rates. Owing to the psychological impact of COVID-19, this review was aimed at examining the pre- and post-COVID-19 turnover and turnover intention rates and their predictors. In this review, we used Whittemore and Knafl’s integrative review method, and evaluated the primary resources by using a modified Johns Hopkins Nursing Evidence-Based Practice Research Evidence Appraisal Tool. On the basis of the review of 43 studies, we can conclude that the COVID-19 pandemic has impacted both the rates and predictors of nurses’ turnover intention.

This review emphasized the alarming rates of nurses’ turnover intention, both before and after the COVID-19 pandemic. Most of the reviewed studies identified the predictors of nurses’ turnover intention. Nursing managers and leaders should utilize this knowledge and develop policies and programs to reduce the impacts of the predictors that increase nurses’ turnover intention and enhance the predictors that increase nurses’ retention. For example, many studies have identified leadership style as one of the predictors of turnover. Thus, new orientation and development programs for managers and leaders must be mandated to reduce staff turnover intention.

Owing to the COVID-19 pandemic, nursing staff have been experiencing increased stress, anxiety, and fear of contracting the infection. Improving nurses’ competencies in caring for patients with COVID-19 might reduce these psychological factors, thereby reducing turnover intention. Moreover, ensuring the availability of personal protective equipment might reassure nurses and reduce their fear of the disease. Nursing managers and leaders should also provide psychological support and counseling to nursing staff during the COVID-19 pandemic to reduce their turnover intention.

## Figures and Tables

**Table 1:** Inclusion criteria.

Criteria
Primary reports of studies that utilized any research method (qualitative, quantitative, or mixed method)
Articles published between 2016 and May, 2021
Articles published in academic journals “peer reviewee process”
Articles published in English language
The articles’ full text available through the reviewed databases
Examine or discuss turnover or turnover intention among nurses

**Table 2:** Summary of quality assessment of the reviewed articles (*n* = 43).

Appraisal Item	Yes	No
1. Does the researcher identify what is known and not known about the problem and how the study will address any gaps in knowledge?	43	0
2. Was the purpose of the study clearly presented?	43	0
3. Was the literature review current (most sources within last 5 years or classic)?	19	24
4. Was sample size sufficient based on study design and rationale?	43	0
5. Are data collection methods described clearly?	41	2
6. Were the instruments reliable (Cronbach’s α [alpha] >0.70)?	36	7
7. Was instrument validity discussed?	25	18
8. If surveys/questionnaires were used, was the response rate >25%?	36	7
9. Were the results presented clearly?	43	0
10. If tables were presented, was the narrative consistent with the table content?	43	0
11. Were study limitations identified and addressed?	40	3
12. Were conclusions based on results?	43	0
Overall quality rating High 41 Good 2 Low 0		

From 12 to 9 high quality; from 8 to 5 good quality; from 4 to 0 low quality.

**Table 3:** Pre- and post-COVID-19 nurses’ turnover literature comparison.

Number	Author/ Year	Purpose	Post COVID-19 Pandemic?	Design, Sample, & Setting	Variable, Definition & Measurement	Prevalence of Turnover	Predictors of Turnover Intention
1	Chen et al., 2021 [5]	Investigate nurses’ competence in nursing care, clinical stress, and intention to stay in their current organization.	Yes	Cross-sectional design; data was collected between March and May of 2020; 333 novice nurses participated (response rate = 83.25%).	Variable: Intention to stay Definition: Intentions of nurses to remain in their current positions Measure: The stay in the nursing workplace scale (a four-point Likert scale: 1 = strongly, 4 = strongly agree). A high average score indicated a low willingness to stay.	The participants’ average score of willingness to stay in their job was 2.00 ± 0.46	Rate of providing care to COVID-19 patients, taken a course on caring for COVID-19 and prevention after starting work, willingness to provide services, and clinical stress
2	Khattak et al., 2020 [16]	Examine the effect of COVID-19 fear on nurses’ turnover intention, secondary trauma, and psychological distress; explore the possible moderating role of leadership support	Yes	Cross-sectional design; convenience sampling; 380 nurses participated (response rate of 54.28%).	Variable: Turnover intention Definition: NA Measure: Labrague et al., turnover intention scale	The mean turnover intention = 3.38 (SD = 0.985)	Fear of COVID-19, secondary trauma, & psychological distress
3	Kim et al., 2020 [7]	Explore social support effect on job engagement and job retention intention among nurses during the COVID-19 pandemic	Yes	Quantitative design; Data were collected from 11–24 May 2020, 377 nurses participated	Variable: Job retention intention Definition: NA Measure: Ajzen’s questions from the Theory of Planned Behavior. It is composed of a five-point Likert scale, and in this study, the higher the score, the greater the nurse’s intention retain the job	The average job retention intention was 3.909 (SD = 1.004)	Age, work experience, experience in nursing COVID-19 patients, working in COVID-19 divisions, category and type of social support
4	Labrague et al., 2020 [15]	To examine the relative influence of fear of COVID-19 on nurses’ psychological distress, work satisfaction and intent to leave their organization and the profession.	Yes	Cross-sectional design; 261 frontline nurses in five hospitals in the Philippines participated (responses rate 87%).	Variable: Organizational and professional turnover intentions Definition: NA Measure: O’Driscoll & Beehr Two single-items of organizational and professional turnover intention were used; each item was rated on a Likert scale ranging from 1 = strongly disagree to 5 = strongly agree.	The mean for the organizational and professional turnover intention were 1.86 (SD: 1.26) and 2.23 (SD: 1.26), respectively	Fear of COVID-19
5	Liaqat et al., 2021 [19]	Evaluate the rate of workplace bullying and its relationship with nurses’ intent to leave the job	Yes	Cross-Sectional design, data were collected from September 2019 to April 2020; A convenience sample of 242 nurses working in two public and two private teaching hospitals of Lahore participated in the study.	Variable: Turnover intention Definition: A conscious and premeditated decision to leave the organization. Measure: Turnover intention measure using two-items scored from 0 to 7	Turnover intention among nurses affirmed work-related bullying (59.5%); among nurses affirmed person-related bullying (39.7%); among nurses affirmed physically intimidating bullying (40.9%)	Workplace bullying
6	Irshad et al., 2021 [18]	Examine the moderating influence of an ideological contract on the link between psychological anxiety and turnover intention, and to evaluate the impact of perceived COVID-19 danger on nurses’ turnover intentions via the underlying mechanism of psychological anxiety.	Yes	Cross-sectional design; 117 Pakistani nurses treating COVID-19 patients; snowball nonprobability sampling technique was used	Variable: Nurses’ turnover intention Definition: NA Measure: Vigoda three-items Nurses’ turnover intention due to COVID-19; A five-point Likert scale ranging from 1 = strongly disagree to 5 = strongly agree	The mean turnover intention = 3.42 (SD = 0.84)	Perceived threat of COVID-19
7	Nashwan et al., 2021 [20]	Compare nurses’ turnover intentions before and during COVID-19.	Yes	Cross-sectional design; convenience sample of nurse working in Qatar; data were collected between August and September 2020; A total of 512 nurses Participated (response rate = 4.26)	Variable: Turnover Intention Definition: NA Measure: The Roodt’s Turnover Intention Scale (TIS-6); five points scale (range = 1.00–5.00)	turnover intentions increased significantly during COVID-19 (from average of 13.24 to 15.54).	**Before COVID-19:** Age, marital status, years of experience, stress level **During COVID-19:** Marital status, years of experience, deployment, stress level
8	Said & El-Shafei, 2021 [12]	Identify occupational stress, work satisfaction, and intention to leave among nurses caring for probable COVID-19 patients.	Yes	Comparative cross-sectional study; 210 nurses from Zagazig Fever Hospital (ZFH; COVID-19 Triage hospital) versus 210 nurses from Zagazig General Hospital (ZGH; neither triage nor isolation hospital); data collected from 10th to 24th of April 2020.	Variable: Intention to leave Definition: NA Measure: Two items from Lagerlund et al.	Significant difference between intention to leave current position (ZFH =4 0.0%, ZGH = 30.5%); intention to leave current organization (ZFH = 45.2%, ZGH = 34.3%); intend to leave the field of nursing (ZFH = 24.8%, ZGH = 10.0%)	Type of hospital and its related workload
9	De los Santos & Labrague 2020 [21]	Assess fear of COVID-19 among nurses in a community setting.	Yes	Cross-sectional design; 385 nurses participated (response rate of 96.25%)	Variable: Organizational and professional turnover intention Definition: NA Measure: Two single-item measures assessing organizational and professional turnover intention; using a five-point Likert scale ranging from 1 = strongly disagree to 5 = strongly agree	Organizational turnover intention (M = 2.82, SD = 1.21); professional turnover intention (M = 2.87, SD = 1.19)	Nurses’ fear of COVID-19
10	Yañez et al., 2020 [22]	Explore the anxiety, distress, and turnover intention of healthcare workers in Peru during the COVID-19 pandemic.	Yes	Cross-sectional study; data were collected from April 10, 2020 to May 2, 2020; surveyed 400 healthcare workers in 15 of the 24 provinces in Peru; 303 responded to the survey (response rate of 75%)	Variable: Turnover intention Definition: Chance of quitting their present employment Measure: Metcalf et al., two-item turnover intention scale	NA	Younger workers; healthcare workers in the private sector
11	Albougami et al., 2020 [23]	Investigate the impact of job satisfaction and quality of life on nurses’ intention to resign, and investigate the factors that influence the intention of Saudi nurses to quit their existing positions.	No	Cross-sectional study; sample of 318 nurses working in two hospitals in Saudi Arabia. Data was collected between April and May 2018.	Variable: Turnover intention Definition: NA Measure: The Roodt’s Turnover Intention Scale (TIS-6); five points scale (range = 1.00–5.00)	The mean turnover intention of the nurses was 2.91 (SD = 0.81)	Martial status, nationality, clinical area, salary, emotional exhaustion, personal accomplishments, physical health, and psychological health
12	Ayalew & Workineh 2020 [24]	Examine nurses’ intentions to leave their jobs and the factors that influence them in Bahir Dar, Northwest Ethiopia, in 2017.	No	Cross-sectional study; conducted between 1st March to 30th March 2017; simple random sampling was used to select 210 participants	Variable: Intention to leave Definition: An employee’s intention to leave his or her current workplace in order to pursue another employment in the near future Measure: Mark C Hand tool. Seven items with a five-point Likert scale	64.9% (95% CI: [57.6, 71.2]) of the participants have intention to leave their job; 53.4% of them had a high level of intention to leave their job	Work itself and recognition at work
13	Chen et al., 2019 [25]	Examine the impact of the patient–nurse ratio on nurses’ intentions to quit, considering the mediating roles of burnout and job dissatisfaction.	No	Two pooled cross-sectional surveys; data collected in 2013 and 2014; total of 1409 full-time nurses in medical & surgical wards of 24 hospitals in Taiwan participated (response rate = 59.2%).	Variable: Intention to leave Definition: NA Measure: Two items developed for the study; score range of 0 to 100	The average intention to leave among the participants = 37.3 (SD = 26.4)	Standardized Patient–nurse ratio predicted nurses’ intention to leave through nurses’ personal burnout, client-related burnout, and job dissatisfaction
14	de Oliveira et al., 2017 [26]	Investigate the factors related to registered nurses’ (RNs’) desire to leave the profession in big public hospitals in Brazil	No	Cross-sectional study; conducted from 2010 to 2011: All RNs at Rio de Janeiro’s 18 largest public hospitals (>150 beds) were invited; the study sample comprised 3229 RNs (82.7% response rate),	Variable: Intention to leave profession Definition: NA Measure: Single item measured at five points Likert-type scale	22.1% of the participants indicated their intention to leave the profession	Gender, age, not holding a leadership position, highly demanding work, passive work, effort–reward imbalance, poor self-rated health, overcommitment to the job, & poor supervisor support
15	Diehl et al., 2020 [3]	Examine, within the context of burden due to quantitative job demands, the buffering effect of individual, social and organizational resources on nurses’ health and intention to leave.	No	Cross-sectional study was carried out in 2017 among nurses in palliative care in Germany. A total of 1360 nurses responded to the questionnaire (response rate 38.7%)	Variable: Intention to leave the profession Definition: NA Measure: Single-item response categories: never, a few times a month, once or twice a week, three to five times a week and every day	NA	Higher quantitative demands, resources degree of freedom, meeting relatives after death of patients, recognition from supervisor and possibilities for development’
16	Falatah & Conway 2019 [27]	Investigate the relationship between relational coordination, job satisfaction, affective commitment & turnover intention	No	Cross-sectional design; a total of 180 nursesparticipated in the study.	Variable: Turnover intention Definition: NA Measure: The Roodt’s Turnover Intention Scale (TIS-6); five points scale (range = 1.00–5.00)	Turnover intention mean = 3.08 (SD = 0.75)	Nationality, affective commitment, job satisfaction, relational coordination
17	Gabel Shemueli et al., 2016 [11]	Assess the mediating effects of burnout and engagement on the relationship between work characteristics & turnover intentions in the nursing community of two Ibero-American countries.	No	Quantitative study; the sample consists of 316 RNs employed in Uruguay and 502 employed in Spain; the survey was open for one year (from January to December 2012).	Variable: Turnover Intention Definition: NA Measure: Arsenault, Dolan, &Van Ameringen Turnover Intention Scale, consisted of three items rated on a five-point Likert scale (1 = totally disagree, 5 = totally agree)	The mean turnover intention Uruguay nurses = 2.65 (SD = 1.17); Spain nurses = 2.55 (SD = 1.24)	Work overload, burnout, social support, and work engagement.
18	Gebregziabher et al., 2020 [1]	Investigate the association between nurses’ job satisfaction and turnover intention in Axum Comprehensive and Specialized Hospital Tigray, Ethiopia	No	Institution based cross-sectional design; systematic random sampling was used to enroll a total of 148 nurses; the study was conducted from January 2018 to June, 2019	Variable: Turnover intention Definition: Probability that an employee will permanently leave his or her current employer in the near future Measure: Three items developed for the study and had three response options: (1) No; (2) Not sure; and (3) Yes.	64.9% of the participants have intention to leave the organization	Job satisfaction
19	Hsieh et al., 2019 [28]	Examine the link between workplace bullying, mental health, and intention to leave among nurses, as well as the function of self-efficacy as a moderator.	No	Cross-sectional study was conducted from October to December 2016; a total of 550 nurses were invited to participate; a total of 442 participants returned their questionnaires, (response rate = 80.4%).	Variable: Intention to leave Definition: NA Measure: The five-item Employee’s Turnover Intentions and Job Destination Choices Scale; rated on a five-point Likert scale ranging from 1—strong disagreement to 5—strong agreement. The total scores were 5–25, with a higher score indicating a greater intention to leave	The mean intention to leave = 12.82 (SD = 3.58)	Bullying, self-efficacy
20	Kaddourah et al., 2018 [29]	Evaluate the quality of nursing work life (QNWL), investigate nurses’ turnover intentions, and investigate the relationship between QNWL and nurses’ turnover intention.	No	Cross-sectional study; two hospitals selected randomly from Riyadh, Saudi Arabia; from March 2015 to March 2016; Nurses working at different shifts were selected randomly from the two hospitals by a quota-based sample, 364 nurses were recruited (response rate = 91%).	Variable: Turnover intention Definition: NA Measure: The 12-item Hinshaw and Atwood Anticipated Turnover Scale (ATS) a seven-point Likert scale ranging from 1 = agree strongly to 7 = disagree strongly’; greater scores reveal a more intent to leave the current job	Almost 94% indicated a turnover intention from their current hospital	Gender, years in current position
21	Ki et al., 2020 [30]	Identify and cluster shift work nurses’ health issues, as well as the relationships between health issues and turnover intention.	No	Cross-sectional study; the sample consisted of novice nurses (204; from October 2018 to January 2019; response rates = 69.4%) and experienced nurses (300; March 2018 to May 2018 response rates = 89.7%)	Variable: Turnover intention Definition: NA Measure: Single item “I plan on staying for the next year”; measured at four points (strongly agree, agree, disagree, or strongly disagree)	22.2% expressed turnover intention	neuropsychological health issues (sleep disturbance, fatigue, and depression).
22	Kilańska et al., 2019 [6]	Examine the link between the work patterns of Polish nurses and the risk of quitting.	No	A quantitative method; conducted in 2008−2011; sample = 1049 (response rate = 90%)	Variable: Risk of quitting Definition: NA Measure: Not specified	NA	Unplanned work schedule; the employer did not respect the nurses’ preferences about when and how they worked; the nurses were not notified of schedule adjustments; They were dissatisfied with the duration of the work shift; they were dissatisfied with the option of taking days off; they were dissatisfied with the capacity to work in the proposed shift; and the amount of working hours per day fell short of their expectations.
23	Kim et al., 2020 [7]	Investigate the relationship between workplace bullying and burnout, professional quality of life, and turnover intention among clinical nurses.	No	Descriptive cross-sectional study; Data were collected from 324 direct patient-care nurses employed in general hospitals in Seoul, Gyeonggi, and Chungnam; Data were collected between 1 July 2018 and 30 September 2018,	Variable: Turnover intention Definition: The tendency to switch jobs or change one’s occupation owing to dissatisfaction with work Measure: Lawler four-questions turnover intention questionnaire; measured at a five-point scale; range from 1 = not at all, to 5 = very much so	The average turnover intention score was 13.12 (SD = 3.63)	Workplace bullying
24	Koch et al., 2020 [14]	Enhance our understanding of the variables that cause people to leave their jobs.	No	Randomized cross-sectional study on young hospital employees in Germany; conducted in September 2017; total of 1337 employees took part in the survey; the response rate was 13% physicians, 18.5%, nurses 7.5%.	Variable: Intentions to leave the profession Definition: NA Measure: Item 33 of the Copenhagen Psychosocial Questionnaire (COPSOQ) “In the last 12 months, how often have you thought about leaving your profession?”; scored at five points	30.9% of the whole sample frequently considered leaving the profession; it was statistically significantly greater for nurses than for physicians (42.2% vs. 28.2%).	Perceived quality of care, job satisfaction.
25	Kwon 2019 [31]	Determine the impact of flexible work system awareness, organizational commitment, and quality of life on turnover intentions among healthcare nurses.	No	Descriptive correlational design; 226 nurses participated in the study; data were collected from 1 September to 1 October 2018	Variable: Turnover intensionDefinition: NA Measure: Lawler turnover intension questionnaire; four items scored on a five-point Likert scale; Higher scores indicated a higher turnover intention.	48.7% indicated turnover intention	Work satisfaction, colleague satisfaction, subjective health, awareness of flexible work systems, organizational commitment, quality of life.
26	Minamizono et al., 2019 [32]	Determine the impact of flexible work system awareness, organizational commitment, and quality of life on turnover intentions among nurses.	No	Secondary data of a cross-sectional study; a total of 1698 nurses, were invited to participate; 441 nurses provided informed consent and returned the self-administered questionnaires (response rate = 26%)	Variable: Intention to Leave Definition: Real employee retention predictor Measure: Three items measured based on a Likert scale from 1 = strongly disagree, to 4 = strongly agree; subsequently divided into binary, disagree, or agree.	79.8% of the participants have intention to leave	being from the younger generation, agreement with the concept of gender division of labor, high job strain, burnout
27	Naburi et al., 2017 [33]	Identify variables related to job decreased job satisfaction and intention to leave among nurses working in HIV prevention of mother-to-child transmission institutions (PMTCT)	No	Quantitative cross-sectional study was conducted in 36 public health facilities in Dar es Salaam, Tanzania, between March and April 2014; 250 nurses were invited 217 participated (response rate = 87%)	Variable: Intentions to leave the current job Definition: NA Measure: Questionnaire developed for the study: “How frequently do you think about leaving your current job?”; the responses were marked on a seven-item Likert scale ranging from 1 = never to 7 = very often.	35% of the sample intended to leave their job	Job stability dissatisfaction, not being recognized by one’s superior, & poor feedback on the overall unit performance
28	Nikkhah-Farkhani & Piotrowski 2020 [10]	Investigate the variables influencing nurse turnover and the variations between Iranian and Polish nurses in this regard.	No	Descriptive cross-sectional study; Poland (*n* = 165) and in Iran (*n* = 200); data was collected between March to May 2019	Variable: Turnover intention Definition: NA Measure: Four-item Turnover Intention Scale	Statistically significant deference in the average turnover intention between Polish nurses (3.23) and Iranian nurses (2.78 out of 5)	Poland: work–family conflict Iran: job satisfaction
29	Özer et al., 2019 [34]	Investigates the connections between nurses’ perceptions of their authentic leadership, intention to resign, and employee performance	No	A convenience sample of nurses working in a public hospital in the city of Yozgat, Turkey (*n* = 500) was used; the data were collected in December 2017 from 189 participants	Variable: Intention to quit Definition: NA Measure: Cammann et al., Intention to Quit Scale; consists of three items; assessed using a five-point Likert-type The (1 = strongly disagree to 5 = strongly agree)	The average intention to quit 2.31 (SD = 1.09)	Authentic leadership
30	Pélissier et al., 2018 [35]	Examine the relationship between female nursing home caregivers’ intention to leave work, working circumstances, and health status.	No	A multicenter cross-sectional survey design; data were collected between October 2009 and September 2010; 1770 caregivers (19.6% RNs, 80.4% nursing assistants) from 105 nursing homes were included; (response rate = 98%)	Variable: Intention to leave Definition: Intention to leave work with the elderly Measure: Not specified	26.3% of the participants wished to leave their work with the elderly: 26.8% nursing assistants and 24.2% RNs	**RNs:** deteriorated care-team or resident relations, & perceived elevated hardship due to the proximity of residents’ death. **Nursing assistants:** deteriorated management relation, with job insecurity and elevated hardship due to the residents’ intellectual deterioration, & impaired physical or psychological health status
31	Qi et al., 2020 [36]	Investigate the consequences of patient maltreatment on nurses’ job satisfaction and turnover intention through work meaningfulness and emotional dissonance, and the moderating impact of hostile attribution bias	No	Three-wave survey; 1200 nurses were asked to participate; 1067 participated in round 1; in round 2 a total of 921 responded; at the final round 657 nurses responded (valid response rate = 54.75%)	Variable: Turnover intention Definition: NA Measure: Knudsen et al., Turnover intention (T3). A three-item scale; seven-point Likert scale; 1 = totally disagree to 7 = totally agree	The average turnover intention = 2.908 (SD = 1.434)	Mistreatment by patients through emotional dissonance.
32	Rahnfeld et al., 2016 [37]	Investigate the relationship of care setting (nursing homes and home care) with geriatric nurses’ intention to leave their job and profession.	No	Cross-sectional study; a sample of 278 RNs and nursing aides in German geriatric care	Variable: Intention to leave job and profession Definition: NA Measure: Simon et al., four-item adapted turnover intention questions and three items from Price Intention to Quit Questionnaire (ITQ); rated with a five-point rating scale	One-fifth to a third of respondents mentioned that they had thought about changing units, institutions, professions, or leaving the labor market entirely several times a year	Demands and resources with job satisfaction as mediator.
33	Sawaengdee et al., 2016 [38]	Identify the rates, patterns, trends, and drivers of work transition, as well as the frequency, incidence, and long-term changes in important health issues among Thai nurses.	No	Longitudinal prospective cohort study comprising multiple age cohorts, started in 2009 and expected to run until 2027; in the first round 18,756 nurses participated (response rate = 58.6%); second round (response rate = 60.2%), last round included 3020 new RN (response rate = 38.3%)	Variable: Intention to leave nursing career Definition: NA Measure: Intension to leave and to return to nursing career	Overall, around 15.4% of nurses reported an intention to leave their nursing career; in the last round, 11.2% reported their intention to leave nursing in the next 2 years.	NA
34	Sharififard et al., 2019 [39]	Determine the relationship between the desire to leave the nursing profession and the work environment and demographic variables.	No	Cross-sectional design; random sampling was used and 206 nurses from six hospitals participated (response rate = 92%)	Variable: Intention to leave the job Definition: NA Measure: Three items related to intention to leave the job rated using a seven-point Likert scale; 1 = strongly disagree to 7 = strongly agree	23.70% of the participants indicated a high level of intention to leave the profession; 25.10% of the participants had moderate intention.	Work climate, type of employment, marital status, and overtime working
35	Sungur et al., 2019 [40]	Explore the link between paternalistic leadership, organizational cynicism, and the desire to resign among nurses.	No	The study population consisted of nurses working in a public hospital in the city of Mersin, Turkey; data were collected in January 2018 from 215 nurses; (response rate = 44%)	Variable: Intention to Quit Job Definition: Intention to quit one’s job refers to a circumstance in which an employee of one organization has contemplated finding employment with another organization owing to discontent with present working conditions. Measure: Cammann et al., intention to quit; consists of a total of three items with a five-point Likert response form 1 = strongly disagree to 5 = strongly agree	Mean Intention to Quit = 1.88 (SD = 0.99)	The dimensions of organizational cynicism and paternalistic leadership
36	Tei-Tominaga et al., 2018 [41]	Investigate the factors influencing the intention to leave among female hospital nurses in a large Japanese sample, divided into four generations based on age, and taking economic situations into account	No	Cross-sectional design; a convenience sampling of nurses from 30 hospitals; out of 11,171 nurses 5763 participated (response rate = 51.6%)	Variable: Intention to Leave Definition: NA Measure: A six-item intention to leave scale; responses were scored on a four-point Likert-type scale, with higher scores representing greater intention to leave	Mean intention to leave based of the participants generation: 1980s (14.53 ± 4.83), after 1990 (13.65 ± 5.00), between 1965 and 1979 (13.47 ± 4.80), and between 1950 and 1964 (12.88 ± 4.53)	Having children increased intention to leave in the generation born in 1965–1979, having family members in need of caregiving other than children decreased the risk in the generation born in the 1980s
37	Van der Heijden et al., 2019 [42]	Explore if burnout as a result of the combined influence of perceived effort and job meaning modulates the link with occupational turnover intention	No	Longitudinal study; questionnaire completed twice (1-year time lag) by RNs working in hospitals (63.4%), old peoples’ homes (15.4%), and home care (21.1%) was conducted; the final sample comprised 1187 nurses; first measurement response rate for = 43.6%; second time = 29.5%.	Variable: Occupational Turnover intention Definition: NA Measure: Hasselhorn, Tackenberg, and Mueller’s three-item scale; a five-point rating scale ranging from: 1 = never, to 5 = every day	Nurses’ mean intention for occupational turnover was 1.43 (SD = 0.7)	Higher burnout levels appeared to lead to a higher occupational turnover intention.
38	Vévoda et al., 2016 [2]	Identify work-environment elements that are essential to general nurses when deciding whether or not to quit their current workplace	No	An observational and a cross-sectional study; 2223 nurses working in 74 hospitals and 23 healthcare institutions in the Czech Republic were invited; 1992 nurses were interviewed between 2011 and 2012	Variable: Turnover intention Definition: A clear and determined desire to quit an organization Measure: Single item “Yes, I am going to leave my employer if a good opportunity arises.”; with two options “I do not know, I have not decided yet.” “No, I am going to stay with my employer even if a good opportunity arises.”	34.7% of nurses would leave their current employer	Salary, availability of modern technology and instruments, and social benefits provided by the employer.
39	Wang et al., 2020 [43]	Examine the links between work satisfaction, burnout, and turnover intention, as well as the predictors of turnover intention, with the goal of keeping primary care practitioners (PCPs) in rural China.	No	Multistage cluster sampling method; a cross-sectional survey conducted in Shandong Province, China; December 2017; sample = 1148 PCPs (response rate = 82%)	Variable: Turnover intention Definition: The process through which workers depart an organization to pursue other opportunities. Measure: Single Likert item: “Do you have the thoughts of leaving this faculty for other jobs elsewhere at present?”, with their responses being rated from 1 = highly disagree to 5 = highly agree	14.06% of the respondents had high turnover intention.	Work environment satisfaction, medical practicing environment satisfaction, and organizational management reduced personal accomplishment
40	Wubetie et al., 2020 [44]	Evaluate the intention of nurses to leave emergency departments and associated variables in selected governmental hospitals in Addis Ababa, Ethiopia.	No	Institutional-based cross-sectional study; conducted on 102 nurses in three selected governmental hospitals, Addis Ababa, from 19 February to 31 March 2018; (response rate = 91.1%)	Variable: Turnover intention Definition: Likelihood that an employee will quit the present institution within a particular time frame owing to a variety of variables Measure: Single dictums item	77.5% respondents had intention to leave the current working unit of the emergency department or hospital.	Educational status, monthly income of less than 3145 Ethiopian Birrs, and professional autonomy
41	Yang et al., 2017 [45]	Examine job pressure and other variables influencing nurses’ intentions to leave	No	Cross-sectional study; conducted in 2013 with multistage sampling; 800 RNs with >1 year of work were recruiting; 90% valid responses	Variable: Turnover intention Definition: A psychological propensity to abandon an organization or a job Measure: Dongrong and Jingyuan Scale of Intent to Leave the Profession; the scale consists of six single-choice questions, directly asking about the respondent’s intension to turnover. Each response was scored in four points; 1 = frequently, and 4 = never; high scores indicate a weak intention to leave the profession	The mean score for turnover intention was (15.00 ± 3.24); 19% = strong/very strong turnover intention, (62%), weak turnover intention and (19%) very weak turnover intention	Age, work pressure, job duty and career commitment
42	Yang & Kim 2016 [46]	Build and test a model of turnover intention among clinical nurses that takes into account the impacts of compassion fatigue, coping, social support, and work satisfaction:	No	Cross-sectional correlational design; participants were 283 clinical nurses in four general hospitals in Korea	Variable: Turnover intention Definition: NA Measure: Park’s tool; consists of four questions rated on a five-point Likert scale	Turnover intention = 3.7 (SD = 0.93)	Job satisfaction.
43	Zaheer et al., 2019 [47]	Investigating how nurses’, allied health professionals’, and clerical workers’ opinions of immediate supervisors, collaboration, and mindful organization influence their desire to leave.	No	Cross-sectional survey conducted at a large community hospital 50 km from central Toronto, Canada; data were collected from nurses, allied health professionals, and unit clerks between 30 September 2015, and 1 February 2016; a total of 185 completed surveys were returned (response rate = 74.1%)	Variable: Turnover intention Definition: An employee’s behavioral desire to leave his or her current work by either transferring to a different unit within the same organization or seeking employment at a different organization while remaining in his or her vocation. Measure: A three-item turnover intention measure using a seven-point Likert scale where a higher score indicated a higher likelihood that a person would quit his/her current job.	The average turnover intention = 3.20 (SD = 1.72)	Staff perceptions of teamwork were the positive effect of supervisory leadership

## Data Availability

Not applicable.

## References

[1] Gebregziabher D., Berhanie E., Berihu H., Belstie A., Teklay G. (2020). The relationship between job satisfaction and turnover intention among nurses in Axum comprehensive and specialized hospital Tigray, Ethiopia. BMC Nurs..

[2] Vévoda J., Vévodová Š., Bubeníková Š., Kisvetrová H., Ivanová K. (2016). Datamining techniques—Decision tree: New view on nurses’ intention to leave. Cent. Eur. J. Nurs. Midwifery.

[3] Diehl E., Rieger S., Letzel S., Schablon A., Nienhaus A., Pinzon L.C.E., Dietz P. (2020). Health and intention to leave the profession of nursing—Which individual, social and organisational resources buffer the impact of quantitative demands? A cross-sectional study. BMC Palliat. Care.

[4] Yang H., Lv J., Zhou X., Liu H., Mi B. (2017). Validation of work pressure and associated factors influencing hospital nurse turnover: A cross-sectional investigation in Shaanxi Province, China. BMC Health Serv. Res..

[5] Chen H.-M., Liu C.-C., Yang S.-Y., Wang Y.-R., Hsieh P.-L. (2021). Factors Related to Care Competence, Workplace Stress, and Intention to Stay among Novice Nurses during the Coronavirus Disease (COVID-19) Pandemic. Int. J. Environ. Res. Public Health.

[6] Kilańska D., Gaworska-Krzemińska A., Karolczak A., Szynkiewicz P., Greber M. (2019). Work patterns and a tendency among Polish nurses to leave their job. Med. Pract..

[7] Kim Y.-J., Lee S.-Y., Cho J.-H. (2020). A Study on the Job Retention Intention of Nurses Based on Social Support in the COVID-19 Situation. Sustainability.

[8] Falatah R., Salem O.A. (2018). Nurse turnover in the Kingdom of Saudi Arabia: An integrative review. J. Nurs. Manag..

[9] Perry S.J., Richter J.P., Beauvais B. (2018). The Effects of Nursing Satisfaction and Turnover Cognitions on Patient Attitudes and Outcomes: A Three-Level Multisource Study. Health Serv. Res..

[10] Nikkhah-Farkhani Z., Piotrowski A. (2020). Nurses’ turnover intention a comparative study between Iran and Poland. Med. Pract..

[11] Shemueli R.G., Dolan S.L., Ceretti A.S., del Prado P.N. (2016). Burnout and Engagement as Mediators in the Relationship between Work Characteristics and Turnover Intentions across Two Ibero-American Nations. Stress Health.

[12] Said R.M., El-Shafei D.A. (2020). Occupational stress, job satisfaction, and intent to leave: Nurses working on front lines during COVID-19 pandemic in Zagazig City, Egypt. Environ. Sci. Pollut. Res..

[13] World Health Organization (2021). COVID-19 Weekly Epidemiological Update 22.

[14] Koch P., Zilezinski M., Schulte K., Strametz R., Nienhaus A., Raspe M. (2020). How Perceived Quality of Care and Job Satisfaction Are Associated with Intention to Leave the Profession in Young Nurses and Physicians. Int. J. Environ. Res. Public Health.

[15] Labrague L.J., Santos J.A.A. (2020). Fear of COVID-19, psychological distress, work satisfaction and turnover intention among frontline nurses. J. Nurs. Manag..

[16] Khattak S.R., Saeed I., Rehman S.U., Fayaz M. (2020). Impact of Fear of COVID-19 Pandemic on the Mental Health of Nurses in Pakistan. J. Loss Trauma.

[17] Whittemore R., Knafl K. (2005). The integrative review: Updated methodology. J. Adv. Nurs..

[18] Irshad M., Khattak S.A., Hassan M.M., Majeed M., Bashir S. (2020). Withdrawn: How perceived threat of Covid-19 causes turnover intention among Pakistani nurses: A moderation and mediation analysis. Int. J. Ment. Health Nurs..

[19] Liaqat M., Liaqat I., Awan R., Bibi R. (2021). Exploring Workplace Bullying and Turnover Intention among Registered Nurses in Tertiary Hospitals, Lahore, Pakistan. Int. J. Nurs. Educ..

[20] Nashwan A.J., Abujaber A.A., Villar R.C., Nazarene A., Al-Jabry M.M. (2021). The impact of COVID-19: A comparison of Nurses’ turnover intentions before and during the COVID-19 pandemic in Qatar. J. Pers. Med..

[21] De los Santos J.A.A., Labrague L.J. (2020). Impact of COVID-19 on the Psychological Well-Being and Turnover Intentions of Frontline Nurses in the Community: A Cross-Sectional Study in the Philippines. medRxiv.

[22] Yáñez J.A., Jahanshahi A.A., Alvarez-Risco A., Li J., Zhang S.X. (2020). Anxiety, Distress, and Turnover Intention of Healthcare Workers in Peru by Their Distance to the Epicenter during the COVID-19 Crisis. Am. J. Trop. Med. Hyg..

[23] Albougami A.S., Almazan J.U., Cruz J.P., Alquwez N., Alamri M.S., Adolfo C., Roque M.Y. (2020). Factors Affecting Nurses’ Intention to Leave Their Current Jobs in Saudi Arabia. Int. J. Health Sci..

[24] Ayalew E., Workineh Y. (2020). Nurses’ intention to leave their job and associated factors in Bahir Dar, Amhara Region, Ethiopia, 2017. BMC Nurs..

[25] Chen X., Ran L., Zhang Y., Yang J., Yao H., Zhu S., Tan X. (2019). Moderating role of job satisfaction on turnover intention and burnout among workers in primary care institutions: A cross-sectional study. BMC Public Health.

[26] de Oliveira D.R., Griep R.H., Portela L.F., Rotenberg L. (2017). Intention to leave profession, psychosocial environment and self-rated health among registered nurses from large hospitals in brazil: A cross-sectional study. BMC Health Serv. Res..

[27] Falatah R., Conway E. (2018). Linking relational coordination to nurses’ job satisfaction, affective commitment and turnover intention in Saudi Arabia. J. Nurs. Manag..

[28] Hsieh Y.-H., Wang H.-H., Ma S.-C. (2019). The mediating role of self-efficacy in the relationship between workplace bullying, mental health and an intention to leave among nurses in Taiwan. Int. J. Occup. Med. Environ. Health.

[29] Kaddourah B., Abu-Shaheen A.K., Al-Tannir M. (2018). Quality of nursing work life and turnover intention among nurses of tertiary care hospitals in Riyadh: A cross-sectional survey. BMC Nurs..

[30] Ki J., Ryu J., Baek J., Huh I., Choi-Kwon S. (2020). Association between Health Problems and Turnover Intention in Shift Work Nurses: Health Problem Clustering. Int. J. Environ. Res. Public Health.

[31] Kwon M. (2019). Effects of recognition of flexible work systems, organizational commitment, and quality of life on turnover intentions of healthcare nurses. Technol. Health Care.

[32] Minamizono S., Nomura K., Inoue Y., Hiraike H., Tsuchiya A., Okinaga H., Illing J. (2019). Gender Division of Labor, Burnout, and Intention to Leave Work Among Young Female Nurses in Japan: A Cross-Sectional Study. Int. J. Environ. Res. Public Health.

[33] Naburi H., Mujinja P., Kilewo C., Orsini N., Bärnighausen T., Manji K., Biberfeld G., Sando D., Geldsetzer P., Chalamila G. (2017). Job satisfaction and turnover intentions among health care staff providing services for prevention of mother-to-child transmission of HIV in Dar es Salaam, Tanzania. Hum. Resour. Health.

[34] Özer Ö., Uğurluoğlu Ö., Sungur C., Çirakli Ü. (2019). The Relationship Between Authentic Leadership, Performance and Intention to Quit the Job of Nurses. Hosp. Top..

[35] Pélissier C., Charbotel B., Fassier J.B., Fort E., Fontana L. (2018). Nurses’ Occupational and Medical Risks Factors of Leaving the Profession in Nursing Homes. Int. J. Environ. Res. Public Health.

[36] Qi L., Wei X., Li Y., Liu B., Xu Z. (2020). The Influence of Mistreatment by Patients on Job Satisfaction and Turnover Intention among Chinese Nurses: A Three-Wave Survey. Int. J. Environ. Res. Public Health.

[37] Rahnfeld M., Wendsche J., Ihle A., Müller S.R., Kliegel M. (2016). Uncovering the care setting–turnover intention relationship of geriatric nurses. Eur. J. Ageing.

[38] Sawaengdee K., Tangcharoensathien V., Theerawit T., Thungjaroenkul P., Thinkhamrop W., Prathumkam P., Chaichaya N., Thinkhamrop K., Tawarungruang C., Thinkhamrop B. (2016). Thai nurse cohort study: Cohort profiles and key findings. BMC Nurs..

[39] Sharififard F., Asayesh H., Rahmani-Anark H., Qorbani M., Akbari V., Jafarizadeh H. (2019). Intention to leave the nursing profession and its relation with work climate and demographic characteristics. Iran. J. Nurs. Midwifery Res..

[40] Sungur C., Özer Ö., Saygili M., Uğurluoğlu Ö. (2019). Paternalistic Leadership, Organizational Cynicism, and Intention to Quit One’s Job in Nursing. Hosp. Top..

[41] Tei-Tominaga M., Asakura K., Asakura T. (2018). Generation-common and-specific factors in intention to leave among female hospital nurses: A cross-sectional study using a large Japanese sample. Int. J. Environ. Res. Public Health.

[42] Van Der Heijden B., Mahoney C.B., Xu Y. (2019). Impact of Job Demands and Resources on Nurses’ Burnout and Occupational Turnover Intention Towards an Age-Moderated Mediation Model for the Nursing Profession. Int. J. Environ. Res. Public Health.

[43] Wang H., Jin Y., Wang D., Zhao S., Sang X., Yuan B. (2020). Job satisfaction, burnout, and turnover intention among primary care providers in rural China: Results from structural equation modeling. BMC Fam. Pract..

[44] Johnson M. (2020). Wuhan 2019 Novel Coronavirus—2019-nCoV. Mater. Methods.

[45] Wubetie A., Taye B., Girma B. (2020). Magnitude of turnover intention and associated factors among nurses working in emergency departments of governmental hospitals in Addis Ababa, Ethiopia: A cross-sectional institutional based study. BMC Nurs..

[46] Yang Y.H., Kim J.K. (2016). Factors Influencing Turnover Intention in Clinical Nurses: Compassion Fatigue, Coping, Social Support, and Job Satisfaction. J. Korean Acad. Nurs. Adm..

[47] Zaheer S., Ginsburg L., Wong H.J., Thomson K., Bain L., Wulffhart Z. (2019). Turnover intention of hospital staff in Ontario, Canada: Exploring the role of frontline supervisors, teamwork, and mindful organizing. Hum. Resour. Health.

